# Estrogen Receptor Hormone Agonists Limit Trauma Hemorrhage Shock-Induced Gut and Lung Injury in Rats

**DOI:** 10.1371/journal.pone.0009421

**Published:** 2010-02-25

**Authors:** Danielle Doucet, Chirag Badami, David Palange, R. Paul Bonitz, Qi Lu, Da-Zhong Xu, Kolenkode B. Kannan, Iriana Colorado, Rena Feinman, Edwin A. Deitch

**Affiliations:** Department of Surgery, University of Medicine & Dentistry of New Jersey (UMDNJ)-New Jersey Medical School, Newark, New Jersey, United States of America; Pennsylvania State University, United States of America

## Abstract

**Background:**

Acute lung injury (ALI) and the development of the multiple organ dysfunction syndrome (MODS) is a major cause of death in trauma patients. Earlier studies in trauma hemorrhagic shock (T/HS) have documented that splanchnic ischemia leading to gut inflammation and loss of barrier function is an initial triggering event that leads to gut-induced ARDS and MODS. Since sex hormones have been shown to modulate the response to T/HS and proestrous (PE) females are more resistant to T/HS-induced gut and distant organ injury, the goal of our study was to determine the contribution of estrogen receptor (ER)α and ERβ in modulating the protective response of female rats to T/HS-induced gut and lung injury.

**Methods/Principal Findings:**

The incidence of gut and lung injury was assessed in PE and ovariectomized (OVX) female rats subjected to T/HS or trauma sham shock (T/SS) as well as OVX rats that were administered estradiol (E2) or agonists for ERα or ERβ immediately prior to resuscitation. Marked gut and lung injury was observed in OVX rats subjected to T/HS as compared to PE rats or E2-treated OVX rats subjected to T/HS. Both ERα and ERβ agonists were equally effective in limiting T/HS-induced morphologic villous injury and bacterial translocation, whereas the ERβ agonist was more effective than the ERα agonist in limiting T/HS-induced lung injury as determined by histology, Evan's blue lung permeability, bronchoalevolar fluid/plasma protein ratio and myeloperoxidase levels. Similarly, treatment with either E2 or the ERβ agonist attenuated the induction of the intestinal iNOS response in OVX rats subjected to T/HS whereas the ERα agonist was only partially protective.

**Conclusions/Significance:**

Our study demonstrates that estrogen attenuates T/HS-induced gut and lung injury and that its protective effects are mediated by the activation of ERα, ERβ or both receptors.

## Introduction

Trauma is the leading cause of death in people under the age of 40 and development of the multiple organ dysfunction syndrome (MODS) is a leading cause of death in trauma patients surviving the initial 72 hour injury period as well as in other intensive care unit patient populations [1]. Although somewhat controversial, the majority of the clinical [2]–[4] and experimental [2], [5] evidence emerging over the last decade suggest that the response to injury, shock and sepsis may differ between males and females, with females being more resistant to the adverse consequences of trauma and sepsis than males. Thus, understanding the mechanisms by which trauma-hemorrhagic shock (T/HS) leads to MODS, as well as the role of sex hormones in modulating this response, is of major potential health importance.

In previous work, utilizing male rats, non-human primates and mini-pigs, we found that T/HS-induced acute lung injury, as well as neutrophil activation, RBC dysfunction, bone marrow suppression and endothelial cell injury and dysfunction were related to gut injury and the release of gut-derived factors into the mesenteric lymphatics rather than the portal vein [6]–[8]. Based on the pioneering work from Dr Chaudry's laboratory showing that sex hormones are important modulators of the response to T/HS [5], [9], we carried out subsequent studies investigating the role of sex hormones in the susceptibility and resistance to T/HS-induced gut injury and gut-induced MODS. The results of this work indicated that female rats were more resistant to T/HS-induced gut injury and did not produce biologically-active mesenteric lymph [10], [11]. Furthermore, we found that sex hormone-related gut protection was associated with abrogation of T/HS-induced lung injury [12], neutrophil activation [11], RBC dysfunction [13] and bone marrow suppression [14], [15].

In the present study, we hypothesized that estrogen protected against T/HS-induced gut injury, at least in part, by limiting enterocyte iNOS production which in turn prevented gut-induced lung injury. The rationale for studying the effects of estrogen as well as selective estrogen receptor (ER)α and β agonists on enterocyte iNOS induction in T/HS-induced gut injury is based on three major lines of evidence. First, there is a large body of evidence documenting that increased iNOS activity is involved in the pathogenesis of ischemia-reperfusion-mediated intestinal injury in a number of model systems [16], [17]. Secondly, our previous observations documented a direct correlation between the magnitude of nitric oxide production and gut injury in both hormonally intact and hormonally-modulated male and female rats subjected to T/HS [12], [18]. Lastly, there is increasing experimental evidence that estrogen's protective effects, in models of T/HS and sepsis, are occuring via a non-genomic mechanism involving the activation of specific ERs and that their cellular distribution varies from tissue-to-tissue [19]–[21]. Our findings demonstrate that the adminstration of estradiol (E2) or the ERβ agonist, and to a lesser degree the ERα agonist, ameliorated T/HS-induced gut and lung injury in ovarectomized (OVX) female rats. Furthermore, treatment with E2 or the ERβ agonist blunted the T/HS-induced ileal iNOS response in OVX female rats. These results further validate the concept that estrogen is a key sex hormone that contributes to resistance to T/HS induced gut and lung injury.

## Materials and Methods

### Experimental Design

The first goal of this study was to examine the role played by the individual estrogen receptors in the protection experienced in female rats during hemorrhagic shock as well as its relationship to multiple-organ failure. In this experiment, both proestrus female (PE) rats and OVX rats were subjected to either laparotomy and controlled hemorrhagic shock (T/HS, 35 mm Hg for 90 minutes) or laparotomy plus sham-shock (T/SS). OVX animals were randomized into 4 groups 1) receiving no drug and acting as the negative control, 2) receiving ERα agonist propyl pyrazole triol (PPT, 5 µg/kg BW), 3) receiving ERβ agonist diarylpropionitrile (DPN, 5 µg/kg BW) or 4) receiving 17β-estradiol (E2, 50 µg/kg BW). Dosing for all drugs used was obtained from previously published studies [22]. Both ER agonists were obtained from Tocris Cookson Incorporated (Ellisville MO) and PPT has been shown to be a sub-type specific ERα agonist with 410-fold specificity for ERα over ERβ, while DPN has a 70-fold specificity for ER β over ERα [23]. The EC_50_ for PPT is approximately 200 pM and for DPN it is 0.85 nm. Thus, based on the EC50 of the ER agonists, the dose chosen would be about 50–100 times this EC50 and thus should be at high enough doses to saturate their respective ER but not so high as to lose specificity. All drugs were dissolved in DMSO (Sigma-Aldrich) and were administered immediately prior to resuscitation during the shock or sham-shock period via intraperitoneal (IP) injection. At 3 hours after the shock or sham-shock period, the animals were sacrificed, following which organs and blood were harvested for study. The small intestine was the first organ system examined. Gut injury was measured using histologic evaluation of the percentage of villi injured. Gut permeability was measured by assessing the mesenteric lymph nodes for bacterial translocation. Lung permeability and pulmonary leukosequestration were measured as markers of organ dysfunction. Lung permeability was quantified by calculating percentage of Evan's Blue dye (EBD) present in the broncho-alveolar lavage fluid (BALF) when compared to the plasma. EBD has been shown to be a good indicator of protein leak within the lung as it binds to albumin. The protein content of the BALF and plasma were measured and the amount of BALF protein was used as a second marker of pulmonary permeability. Pulmonary leukosequestration was utilized as a marker of lung injury by quantifying pulmonary myeloperoxidase (MPO).

Since increased iNOS activity and the production of nitric oxide has been implicated in the pathogenesis of T/HS-induced gut injury [16], [17], the second goal was to investigate the potential modulatory effects of individual estrogen receptors on T/HS-induced iNOS expression within the intestine.

### Animals

Female and OVX female Sprague-Dawley rats (Charles River), weighing 300 to 375 g, were used after a minimum acclimatization period of 5 days. OVX animals were allowed at least 14 days after ovarectomy prior to use. All animals were housed under barrier-sustained conditions and kept at 25°C with 12-hour light/dark cycles. The rats had free access to water and chow (Teklan 22/5 Rodent Diet W-8640, Harlan Teklad). All rats were maintained in accordance with the recommendations of the Guide for the Care and Use of Laboratory Animals. The New Jersey Medical School Animal Care Committee approved all animal protocols.

### Surgical Procedure

Rats were anesthetized with IP sodium pentobarbital (50 mg/kg). Using aseptic techniques, the femoral artery and internal jugular vein were isolated and cannulated with polyethylene (PE-50) tubing and 50-gauge silicone catheter containing 0.1 mL heparinized saline (10 units/mL), respectively. Both catheters remained in place for the duration of the experiment. Next, a 3-cm midline laparotomy (trauma) was performed with exposure of the intestine for 15 minutes, followed by closure with a running 4–0 silk suture. Continuous blood pressure monitoring was achieved via the femoral artery catheter (BP-2 Digital Blood Pressure Monitor, Columbus Instruments). Blood was then withdrawn from the internal jugular vein catheter. The mean arterial pressure was reduced to 35 mm Hg and maintained at this level for 90 minutes by withdrawing or re-infusing shed blood as needed. The animals temperature was maintained during the shock period at approximately 37°C by using an electric heating pad under the surgical platform. At the end of the shock period, animals were resuscitated by re-infusing all of the shed blood, restoring the blood pressure back to pre-shock levels. All treatment drugs (17β estradiol, DPN or PPT] were administered via intra-peritoneal injection just prior to resuscitation with shed blood. The T/SS rats were anesthetized, their vessels cannulated, and underwent a laparotomy, but no blood was withdrawn or infused. All animals remained anesthetized throughout the duration of the experiment.

### Determination of Menstrual Stage

Female rats were periodically screened via vaginal swabbing to determine the stage of the estrus cycle as described by Baker et al [24]. All the non-ovariectomized rats were studied only during the PE stage of the estrus cycle.

### Bacterial Translocation

The mesenteric lymph node (MLN) complex was harvested and the level of translocating bacterial quantified as previously described [25]. Briefly, using sterile technique, the MLN complex was harvested, weighed and homogenized in 0.5 ml of sterile saline. Aliquots were plated onto both blood and MacConkey agar plates. These plates were examined at 24 hours of aerobic incubation at 37°C. The colonies were counted on the plates and recorded as bacteria per gram of tissue.

### Histological Analysis of Gut

After sacrifice, a segment of the terminal ileum was excised and fixed in 10% buffered formalin. After processing, semi-thin (2–4 µm) sections were cut, stained with H&E and examined using light microscopy at 100× magnification. In order to deem a villus injured, there must be microscopic evidence of injury. Injury ranged from submucosal edema at the villous tip to frank necrosis of the villi. A total of five random fields from each animal were examined, and the percentage of villous damage for each animal was determined. All results are expressed as the percentage of villi injured per high-power field. All histologic evaluations were performed in a blinded fashion.

### Lung Permeability

Prior to sacrifice, a reference sample was obtained for protein determination. Immediately following collection, 1 mL of EBD was injected via the internal jugular catheter. After 5 minutes, a 1 mL blood sample was withdrawn from the femoral arterial catheter, centrifuged and serial dilutions of the plasma were made to generate an EBD standard curve. After 20 minutes, the lungs were excised and bronchoalveolar lavage (BAL) was performed. The lungs were instilled with 5 mL of phosphate-buffered saline (PBS), rinsed 3 times with PBS and the BALF was collected and centrifuged to remove any cells or debris. EBD in the BALF was measured spectrophotometrically at 620 nm and the concentration of EBD in the BALF was determined by the standard curve. The percentage of EBD in the BALF relative to EBD in the plasma was determined. As a second marker for lung permeability, the BALF/plasma protein ratio was also measured using a refractometer as previously described [26].

### Myeloperoxidase Assay

MPO activity was assessed in lung tissue as previously described [26] and expressed as activity per gram of lung tissue, with 1 unit of MPO activity being the amount of enzyme that will reduce 1 µmol of peroxide per minute.

### Ileal iNOS Immunohistochemistry

Paraffin blocks containing lung tissue was cut in 5 µm thick sections and iNOS positive cells were observed by immunoperoxidase staining. Lung sections were stained with rabbit anti-iNOS antibody (Santa Cruz Biotechnology) or with control solutions. Controls included buffer alone or nonspecific purified rabbit IgG. Endogenous peroxidase activity was quenched though incubation with 3% peroxide and specific iNOS labeling was detected following incubation with biotinylated anti-rat IgG antibody and avidin-biotin peroxidase complex ABC Kit (Santa Cruz Biotechnology). The slides were then counterstained with hematoxylin (Sigma-Aldrich) and the number of iNOS positive cells was quantified per 100 villi counted. To verify iNOS specificity, several sections were incubated with primary antibody without secondary antibody or secondary antibody without primary antibody.

### Statistics

Bacterial Translocation data was analyzed using χ^2^ analysis with Fisher exact test. All other data was analyzed using ANOVA with the Tukey-Kramer multiple comparison test. Data is expressed as mean ± SD. A *P* value of less than 0.05 was considered significant.

## Results

**Figure 1:**
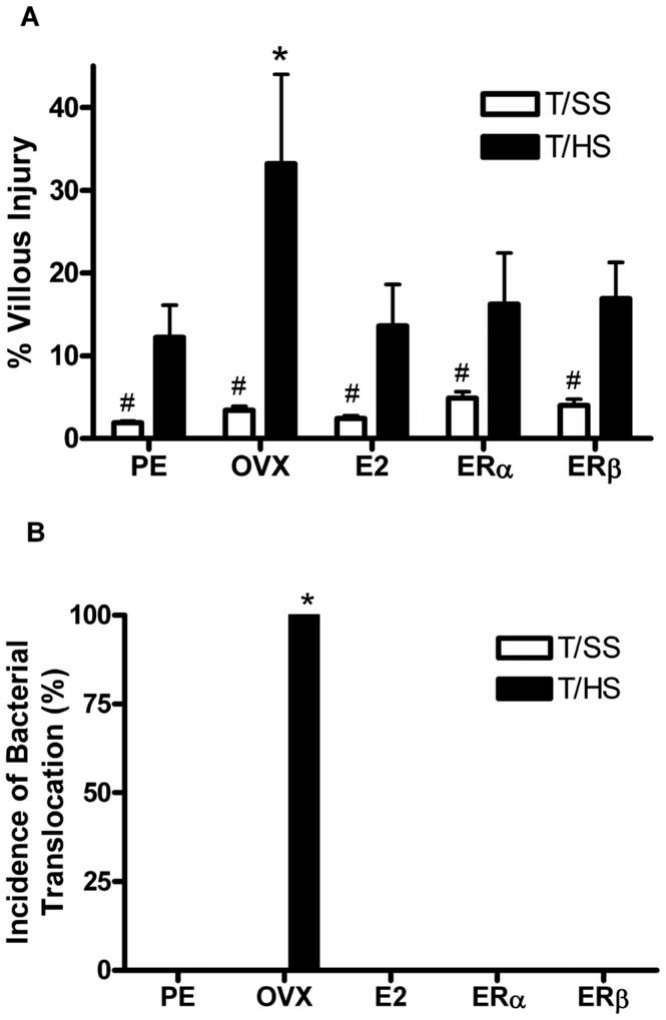
Estradiol and both estrogen receptor agonists ERα and ERβ are protective against T/HS induced gut injury. A) The extent of villus injury 3 hours after T/HS was reduced back to the levels observed in the PE females by all three estrogen-based therapies. A minimum of 200 villi per animal was counted with 6–8 animals per group. *p<0.001 vs all other T/HS groups, #p<0.05 vs all T/HS groups. B) The incidence of bacterial translocation was increased only in the OVX group subjected to T/HS. *p<0.01 vs all other groups with 6–8 animals per group.

As previously reported [18], T/HS caused more gut injury in OVX female rats as compared to PE rats (Figure 1A). The administration of E2 as well the ERα (PPT) and ERβ (DPN) agonists to the OVX female rats at the beginning of reperfusion markedly reduced T/HS-induced villous injury. While all of the T/HS groups did show some morphologic evidence of gut injury when compared to their T/SS counterparts (Figure 1A), only the OVX T/HS group demonstrated bacterial translocation to the MLN complex (2.7×10^3^ and 2.5×10^3^ bacteria/gram of tissue aerobic and enteric respectively) (Figure 1B). Thus, these results suggest that E2 and both ER agonists were able to limit T/HS-induced morphologic gut injury and prevent bacterial translocation.

**Figure 2:**
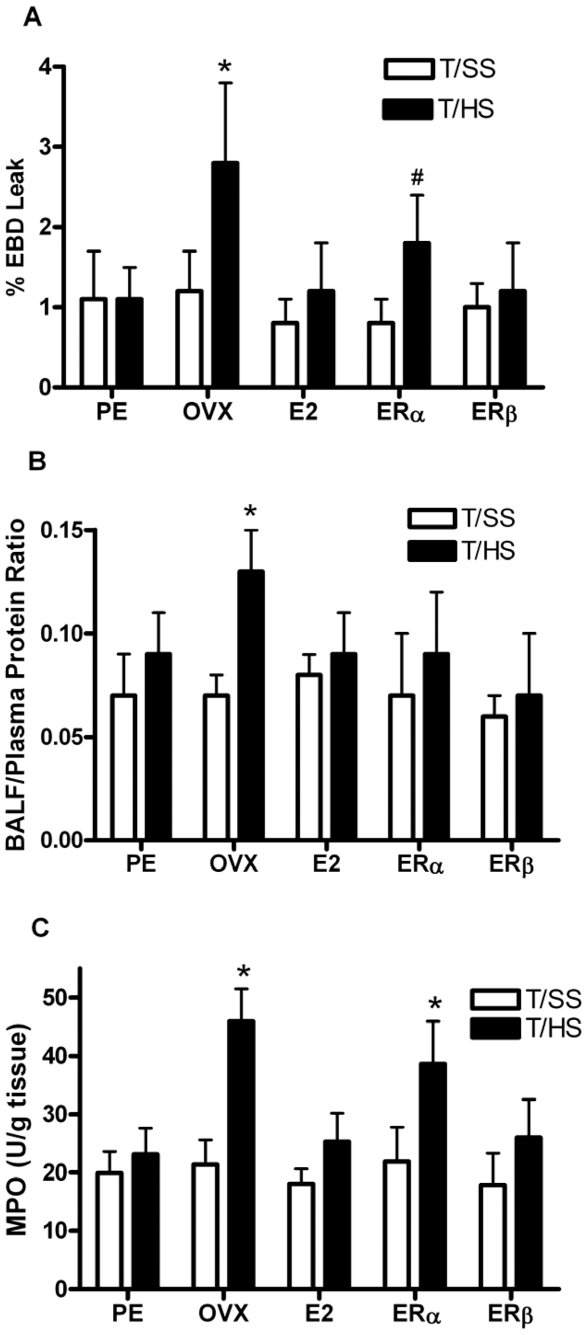
Estradiol and ERβ are more effective than ERα in conferring protection against T/HS induced lung injury. A) T/HS-induced lung injury, as represented by the percentage of EBD leak within the BALF or B) by the BALF/plasma protein ratio, was increased in the ovariectomized rats and reduced by the administration of estradiol of the estrogen receptor agonists. Data expressed as mean ± SD with 6–8 rats per group. *p<0.01 vs all other groups except the ERα T/HS group. #p<0.01 vs ERα T/SS group. C) T/HS increased the degree of lung neutrophil sequestration, as reflected in MPO levels, in the OVX rats and this was abrogated by the administration of E2 and the ERβ but not the ERα agonist. *p<0.05 vs all other groups.

Our earlier studies [18] demonstrated that PE female rats did not develop increased lung permeability after T/HS. As an extension of this observation, we tested the effects of E2 and the ER agonists on T/HS-induced lung permeability. The administration of E2 or the ERβ agonist, DPN, fully prevented an increase in T/HS-induced lung permeability (Figure 2A) whereas the ERα agonist, PPT, was partially protective as compared to their untreated OVX counterparts subjected to T/HS. Similarly, treatment with E2 or PPT, the ERβ agonist, reduced the BALF/plasma protein ratio, a marker for lung permeability (Figure 2B), as well as MPO levels (Figure 2C) in OVX rats subjected to T/HS. DPN, the ERα agonist, was not as effective as E2 and PPT in reducing MPO levels. These results suggest that ERβ is more effective in limiting T/HS-induced lung injury and neutrophil sequestration than ERα.

**Figure 3:**
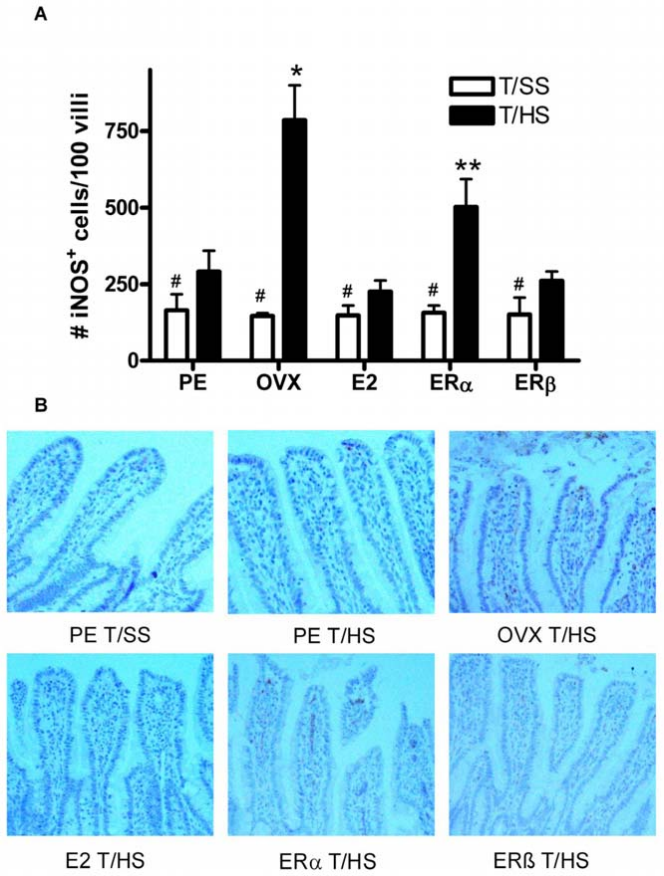
iNOS response is abrogated by both E2 and ERβ. A) The number of iNOS positive enterocytes was increased in all of the T/HS groups but was highest in the OVX rats and the OVX rats receiving the ERα agonist. Data expressed as mean ± SD with a minimum of 100 villi counted per animal with 6–8 animals per group. * p<0.01 vs all other groups, **p<0.01 vs all other groups, and #p<0.05 vs T/HS groups. B) Representative immunohistochemistry micrographs showing iNOS staining of enterocytes.

Since increased iNOS activation has been implicated in the pathogenesis of gut injury after ischemia-reperfusion insults [16], [17], we tested the hypothesis that iNOS would be increased in the enterocytes of OVX but not hormonally intact (PE) female rats after T/HS and that this increased enterocyte iNOS response would be abrogated by the administration of E2. As shown in Figure 3A and 3B, the number of enterocyte iNOS positive cells was increased to a much greater degree in the OVX female rats than the hormonally intact female rats after T/HS. The administration of either E2 or the ERβ agonist completely abrogated the effect of ovariectomy on T/HS-induced enterocyte iNOS expression while the ERα agonist was partially protective.

## Discussion

The biology of estrogen has been extensively studied for decades due to its important reproductive, cardiovascular and other effects. Initially, estrogen was thought to work through a genomic mechanism, where over a period of hours to days, it induces gene transcription and subsequent protein synthesis. However, it now appears that estrogens can also exert their physiologic effects via rapid non-genomic mechanisms occurring within minutes by binding estrogen receptors and activating signaling systems within the cytoplasm [19]. Our observation that the beneficial effects of E2 administration or the administration of the ER agonists on gut and lung function were rapidly observed is consistent with previous studies on the immune system [27], [28] and RBC function [28] as well as organ function [29], [30] suggesting that the beneficial effects of the ER agonists are operating primarily through a non-genomic pathway. Furthermore, because the density of ERα and ERβ varies between organs and among cell populations, these differences could translate into differences in the response to specific estrogen agonists at the tissue and/or cellular levels [20]. For example, similar to our lung results, earlier work from the laboratory of Dr Chaudry has shown that tissues with higher densities of ERβ than ERα, such as the lung, are better protected from T/HS-induced injury by ERβ than ERα agonists [30]. Likewise, this same group has documented that ERα is more important than ERβ in mediating the immuno-protective effects of estrogen on T cells and splenic macrophages as well as liver injury in rats subjected to trauma-hemorrhage [20], [27]. The fact that the intestine contains both ERα and ERβ may help explain why both ER agonists limited T/HS-induced intestinal morphologic injury and bacterial translocation to a similar extent as E2 in the current study.

It has been well documented that splanchnic blood flow decreases out of proportion to the overall decrease in cardiac output in shock states and consequently that the gut is particularly susceptible to ischemia-reperfusion injuries [31]. Based on an increasing body of information, it also seems that the generation of high levels of nitric oxide by iNOS during gut ischemia is involved in the pathogenesis of gut injury [16], [17], [32]. Thus, we tested the concept that T/HS-induced gut injury is related to the combination of the production of excessive levels of iNOS-derived nitric oxide as well as the generation of reactive oxidants and that E2 would limit T/HS-gut and hence gut-induced lung injury, at least in part, by limiting enterocyte iNOS induction. Thus, our observation that hormonally-intact female rats subjected to T/HS had less gut injury and limited induction of enterocyte iNOS as compared to OVX rats supports this concept as does the fact that E2 administration abrogated both the magnitude of gut injury and intestinal iNOS response in OVX rats subjected to T/HS. Furthermore, this observation that E2 limited enterocyte iNOS induction after T/HS is supported by previous studies indicating that intestinal enterocytes contain functional estrogen receptors [33], [34] and that part of estrogen's protective effect on the cardiovascular system occurs via its ability to limit iNOS induction [35], [36]. This notion that increased iNOS activity is a key factor contributing to gut injury and loss of barrier function after T/HS expands upon studies showing that gut injury is greatly reduced in male as well as female iNOS knockout mice subjected to superior mesenteric artery occlusion as compared to their wild-type littermates [32]. Additionally, we and others have documented that nitric oxide directly impairs intestinal barrier function [37], [38] and that LPS-induced enterocyte-derived nitric oxide induces intestinal monolayer permeability in an autocrine fashion [39]. This observation that enterocyte iNOS-generated nitric oxide can promote enterocyte injury in an autocrine fashion highlights the concept that enterocytes can be targets as well as producers of nitric oxide. Our results investigating the ability of E2 and specific ER agonists to directly and specifically limit T/HS-induced enterocyte iNOS induction extends our previous work documenting a gender difference in the plasma nitric oxide and total ileal iNOS responses to T/HS where PE females were found to have a blunted response as compared to males [12], [18]. That is, to our knowledge, the current work shows for the first time that E2 can specifically limit T/HS-induced enterocyte iNOS induction and that this protective effect is primarily being mediated through β estrogen receptor signaling. However, one limitation of this conclusion is that immunohistochemistry is only semi-quantitative as compared to Western blotting analysis. On the other hand, immunohistochemistry does have the advantage that it is specific and can be used to clearly document that iNOS induction was occurring in the enterocytes as opposed to other cells.

Although both ERα and ERβ agonists limited morphologic gut injury and bacterial translocation, the observation that enterocyte iNOS induction after T/HS in the ovariectomized rats was abrogated to a greater extent by the ERβ than the ERα agonist raises an important issue. That is, this observation suggests that the mechanism by which the ERα agonist limited gut injury must involve protective pathways or cell populations independent of its ability to limit the enterocyte iNOS response. One possible explanation is that a significant portion of the ERα agonist's protective effect is being mediated through non-enterocyte cell populations, such as endothelial and immune cells. This possibility is consistent with the fact that both the endothelium [40] and immune cells [27] are more responsive to ERα than ERβ agonists and the intestinal blood flow is better preserved [41], [42] in female than male rats after T/HS. Additionally, ERα agonists have been shown to limit T/HS-induced immune cell activation better than ERβ agonists [27]. Since gut injury after T/HS is multifaceted and involves changes in microcirculatory blood flow, an intestinal inflammatory response as well as enterocyte apoptosis and injury, it is possible that estrogen's protective effects on different components of this process may be transduced via different estrogen receptors. Clearly, further work is needed to sort out this question. In this context, it is important to stress that one limitation of this study was the fact that this was purely a pharmacologic study and that only one dose of each drug was used. Specifically, although the ER agonists are proposed to be highly selective and the doses used in the current study were supported by previous studies and the pharmacology of the agents [22], [23], one can never exclude the fact that any drug may have unknown target effects, which can confound data interpretation. Consequently, it would be important to carry out genetic-based studies to complement the pharmacologic work reported here. For example, siRNA or antisense knockdown approaches as well as the use of ERα and/or ERβ knockout mice could be used to validate as well as extend this work. To date, the effects of major trauma or hemorrhagic shock have not been tested in ERα or ERβ knockout mice.

An additional important observation of potential clinical relevance is that E2 as well as the ER agonists were effective when used in a post-treatment regimen and administered at the end of the shock period at the time of fluid resuscitation. It is important to note that a limitation of ovariectomy in investigating the role of estrogen as a protective factor is that ovariectomy causes significant reductions in progesterone, prolactin and DHEA in addition to estrogen [43]. Although estrogen is not the only sex-related hormone altered by ovariectomy, our results showing that the administration of E2 as well as the ER agonists were sufficient in limiting gut and lung injury further validates the notion that estrogen is the key sex hormone responsible for abrogating gut and lung injury as well as enterocyte iNOS induction after T/HS in female rats.

In the aggregate, although the current as well as other preclinical studies generally indicate that estrogen exerts a protective effect on various organ and cellular systems in shock-trauma models, caution must be exercised in directly applying these results to the clinical arena. That is, clinical studies of major trauma patients as well as septic and other ICU patient populations are not as clear in showing clinical benefit for females, especially when mortality is used as the primary outcome measure [2], [3], [44]. There are several potential explanations for this fact, including the limitations of preclinical trauma-shock models, such as the fact that all of the animals are healthy and have no pre-exist illnesses or confounding social issues such as alcohol ingestion [44], [45]. Additionally, the immuno-inflammatory response of rodents differ to some degree from those of humans [45] as well as the fact that sex hormone levels rapidly change over time in trauma and other patient groups [46], [47] and these patients frequently develop secondary complications, such as sepsis and other second insults during their hospital course that lead to adverse clinical outcomes. Nonetheless, the current study supports the theory that the magnitude of acute gut and lung injury after T/HS are limited by estrogens and that this protection is mediated at least in part through selective estrogen-receptors acting in a rapid non-genomic fashion.
